# Functional analysis of *UGT201D3* associated with abamectin resistance in *Tetranychus cinnabarinus* (Boisduval)

**DOI:** 10.1111/1744-7917.12637

**Published:** 2018-09-26

**Authors:** Meng‐Yao Wang, Xin‐Yang Liu, Li Shi, Jia‐Lu Liu, Guang‐Mao Shen, Ping Zhang, Wen‐Cai Lu, Lin He

**Affiliations:** ^1^ Key Laboratory of Entomology and Pest Control Engineering, College of Plant Protection Southwest University Chongqing China; ^2^ Academy of Agricultural Sciences Southwest University Chongqing China

**Keywords:** abamectin resistance, prokaryotic expression, RNA interference, *Tetranychus cinnabarinus*, UDP‐glycosyltransferases

## Abstract

Uridine diphosphate (UDP)‐glycosyltransferases (UGTs) are widely distributed within living organisms and share roles in biotransformation of various lipophilic endo‐ and xenobiotics with activated UDP sugars. In this study, it was found that the activity of UGTs in abamectin‐resistant (AbR) strain was significantly higher (2.35‐fold) than that in susceptible strain (SS) of *Tetranychus cinnabarinus*. Further analysis showed that 5‐nitrouracil, the inhibitor of UGTs, could enhance the lethal effect of abamectin on mites. From the previous microarray results, we found an UGT gene (*UGT201D3*) overexpressed in AbR strain. Quantitative PCR analysis showed that *UGT201D3* was highly expressed and more inducible with abamectin exposure in the AbR strain. After silencing the transcription of *UGT201D3*, the activity of UGTs was decreased and the susceptibility to abamectin was increased in AbR strain whereas it was not in SS. Furthermore, *UGT201D3* gene was then successfully expressed in *Escherichia coli*. The recombinant UGT201D3 exhibited α‐naphthol activity (2.81 ± 0.43 nmol/mg protein/min), and the enzyme activity could be inhibited by abamectin (inhibitory concentration at 50%: 57.50 ± 3.54 μmol/L). High‐performance liquid chromatography analysis demonstrated that the recombinant UGT201D3 could effectively deplete abamectin (15.77% ± 3.72%) incubating with 150 μg protein for 6 h. These results provided direct evidence that *UGT201D3* was involved in abamectin resistance in *T. cinnabarinus*.

## Introduction

The carmine spider mite, *Tetranychus cinnabarinus* (Boisduval), is one of the major agricultural pests worldwide, infesting vegetables and many other crops including cotton, maize and tobacco (Guo *et al*., 1998; Cakmak & Baspinar, 2005). Many researchers believe that *T. cinnabarinus* and the two‐spotted mite, *Tetranychus urticae*, are two forms (red and green) of a single species (*T. urticae*) because of their similarity in morphological, biological and molecular characteristics (de Mendonca *et al*., 2011; Auger *et al*., 2013). So far, the control of phytophagous mites has mainly relied on chemical acaricides. However, the high reproductive potential and extremely short life cycle of these mites, result in rapid resistance development to many acaricides often after only a few applications, which is a major problem for the control of *T. cinnabarinus* (Devine *et al*., 2001; Stumpf & Nauen, 2001).

Abamectin is widely used as an insecticidal, acaricidal and nematicidal agent. As a macrocyclic lactones compound, it belongs to the family of avermectins (AVMs) which are derived from the soil organism *Streptomyces avermitilis* (Putter *et al*., 1981). Its target is most likely the γ‐aminobutyric acid chloride channels (GABACls), glutamate‐gated chloride channels (GluCls) and histamine‐gated chloride channels (HisCls) (Sigel & Baur, 1987; Zheng *et al*., 2002; McCavera *et al*., 2007). Even though abamectin is very efficient for the control of pests and mites, unfortunately many insects and mites have developed a high resistance to this pesticide (van Leeuwen *et al*., 2010). Up to now, most documents indicated that resistance against abamectin in insects and mites on one hand was dependent on the expression changes of a diverse set of proteins including metabolic enzymes and P‐glycoproteins (van Leeuwen *et al*., 2010; Riga *et al*., 2014; Xu *et al*., 2016), and on the other, was dependent on the mutations of the related chloride channels (Dermauw *et al*., 2012). Besides these factors, it is believed that some other factors may also contribute to abamectin resistance in arthropods.

Uridine diphosphate (UDP)‐glycosyltransferases (UGTs) are responsible for glycosylation by catalyzing the conjugation of a glycosyl group from an activated nucleotide sugars donor, UDP‐glycoside, with various small hydrophobic molecules, and it widely distributes in animals, plants, bacteria and viruses as a multifunctional protein superfamily (Bock, 2003; Mackenzie *et al*., 2005). The UGT protein structure is mainly divided into two parts: the N‐terminal aglycone substrate binding domain and the C‐terminal UDP‐glycoside binding domain (Magdalou *et al*., 2010). Unlike primary metabolic enzymes such as P450s or carboxylesterases (CarEs) which directly act on the toxin molecule to decrease its biological activity, UGTs work in the secondary metabolic process, adding bulky side groups onto toxic compounds to increase their hydrophilicity and facilitating their excretion from the organism (Ahn *et al*., 2012). The functions of UGT enzymes were well studied in mammals. They work as membrane‐bound protein in the lumen of the endoplasmic reticulum utilizing UDP‐glucuronic acid as a sugar donor to conjugate a large number of xenobiotics as well as endobiotics, such as bilirubin and drugs, pollutants, and odorants (Bock, 2003; Bon, 2010). Insect UGT enzymes were similar to plant UGTs, using UDP‐glucose rather than UDP‐glucuronic acid as a sugar donor (Bowles *et al*., 2005). It was reported that many endogenous compounds, like odorant binding proteins and ecdysteroid hormones are glycosylated by UGT enzymes in a range of insect species (Huang *et al*., 2008; Bozzolan *et al*., 2014). It was also reported that insect UGTs play an important role in detoxification of plant allelochemicals, like gossypol, which can partially be metabolized by UGTs via glycosylation in *Helicoverpa armigera* and *Heliothis virescens* (Krempl *et al*., 2016). Some insect UGT genes were up‐regulated in resistant strains and also were suspected to play a role in insecticide‐resistance development, including dichlorodiphenyltrichloroethane (DDT), pyrethroids, carbamates and neonicotinoids resistance (Pedra *et al*., 2004; Vontas *et al*., 2005; Silva *et al*., 2012; Yang *et al*., 2013). Moreover, the over‐expressions of *UGT2* gene in *Leptinotarsa decemlineata* and *UGT2B17* in *Plutella xylostella*, were proved to contribute to imidacloprid‐ and chlorantraniliprole‐resistance formation, respectively (Kaplanoglu *et al*., 2017; Li *et al*., 2017). All of the above studies suggested that the over‐expressions of UGT genes in insects might play important roles in the formation of insecticide resistance, however, the report about the direct interaction between UGT gene products and insecticides is still absent.

In this study, we detected the activity of UGTs in susceptible strain (SS) and abamectin‐resistant (AbR) strain of *T. cinnabarinus*. An UGT gene, *UGT201D3*, was fully sequenced and quantified for its messenger RNA (mRNA) expression profiles in different life stages and both strains, and the transcriptional response of this gene was investigated with exposure to abamectin. The RNA interference (RNAi) method was applied to investigate the role of *UGT201D3* in abamectin resistance in *T. cinnabarinus*, then this gene was successfully expressed in *Escherichia coli* and the enzymatic properties were characterized. High‐perfomance liquid chromatography (HPLC) analysis was performed to confirm the interaction between the recombinant UGT201D3 protein and abamectin.

## Materials and methods

### Mites

Two strains of *T. cinnabarinus* were used in this study: SS, a susceptible strain reared for many years without exposure to insecticides in the laboratory, which was originally collected from a cropland of cowpea in Beibei, Chongqing, China in 1998; AbR, abamectin‐resistant strain, was generated from the SS strain with the selection of abamectin in the laboratory. Detailed information about the acaricide selection process is provided by He *et al*. (2009b). All the mites were cultivated on fresh potted cowpea leaves and kept in an artificial climate chamber at 26 ± 1 °C, 55%–70% relative humidity (RH) and a 14 : 10 h L : D photoperiod.

### Reagents

The 95% abamectin was ordered from Bangnong Chemical Company (Guangzhou, China), 5‐nitrouracil was ordered from Adamas‐beta (Shanghai, China); iQ™ SYBR^®^ Green Supermix was acquired from Bio‐Rad (Hercules, CA, USA); α‐naphthol was ordered from Shanghai Chemical Reagent Company of the Chinese Medical Group (Shanghai, China); pGEM‐T Easy Vector was ordered from Promega (Madison, WI, USA); pET‐28a expression vector and *Eco*R I, *Nde* I were obtained from Takara (Dalian, China). Trans5α and BL21 (DE3) competent cells were acquired from TransGen Biotech (Beijing, China); isopropyl b‐D‐1‐thiogalactopyranoside (IPTG), UDP‐glucose and kanamycin were obtained from Sigma Chemical Co. (St Louis, MO, USA). Other chemicals and reagents were high‐quality commercially available products supplied by local suppliers.

### Determination of UGT activity

The total protein content of the enzyme solution was determined using the Enhanced BCA Protein Assay Kit (Beyotime Biotechnology, China) following the manufacturer's protocol. UGT activity was tested using α‐naphthol as substrate and UDP‐glucose as an activated sugar donor according to the modified method of Real *et al*. (1991) and Krempl *et al*. (2016). The 200 female adult mites (3–5 days old) from different strains were homogenized in 1.5 mL sodiumphosphate buffer (0.1 mol/L, pH 7.8) on ice, then centrifugation at 10 000 × *g* for 15 min at 4 °C, after which the extracted supernatant was tested. Specifically, UDP‐glucose (1 mmol/L), MgCl_2_ (10 mmol/L) and crude enzyme (100 μL) were added to the sodium phosphate buffer (0.1 mol/L, pH 7.8) resulting in a total volume of 500 μL and incubated in 1.5 mL centrifuge tubes for 20 min at 37 °C. Then α‐naphthol (10 μmol/L) was mixed to the incubation mixture conducted at 37 °C for 20 min. Finally, 250 μL of mixture was transferred to the 96‐well plates (Guangzhou JET Bio‐Filtration Products Co., Ltd., Guangzhou, China) with 50 μL of color developing agent (mixed as follows: mass fraction 5% SDS: mass fraction 1% fast blue B salt = 5:2 v/v). After incubation for 10 min at 37 °C, the optical density (OD) value was measured at 600 nm using a microplate reader (EON, BioTek Instruments Inc., Winooski, VT, USA). Control was detected in the absence of UDP‐glucose. The specific activity of UGTs was calculated based on an α‐naphthol standard curve and protein concentration of the enzyme source. All assays were performed in three biological replicates. Data were statistically analyzed by Student's *t*‐test with SPSS 19.0 (SPSS Inc., Chicago, IL, USA); a *P*‐value < 0.05 was considered statistically significant.

### Bioassay

Bioassays of *T. cinnabarinus* were conducted using the modified residual coated vial (RCV) method as described by Feng *et al*. (2011). Abamectin was dissolved in acetone to various concentrations to keep the mortality at 20%–80%, then, thirty 3–5 days old healthy adult females were transferred into the abamectin‐coated centrifuge tube, each dose was repeated three times and the mites treated with acetone were used as control. The mites were checked under anatomical microscope after 24 h treatment. Mites showing immobility or with legs irregularly trembling were considered dead. The 5‐nitrouracil (the specific inhibitor of UGTs) was used to investigate the effect of UGTs on abamectin's toxicity and resistance formation in *T. cinnabarinus* according to relevant studies (Grancharov *et al*., 2001; Li *et al*., 2017). The only difference with the above was that each abamectin solution had 5‐nitrouracil added with a final concentration of 10 mg/L. There were three biological replicates. The median lethal concentrations (LC_50_) were calculated by PoloPlus (Probit and Logit Analysis, LeOra Software, Petaluma, USA).

### RNA Extraction, reverse transcription and cloning of full‐length UGT201D3 cDNA

Total RNA was extracted from 200 female adults (3–5 days old) of *T. cinnabarinus* using the RNeasy^®^ plus Micro Kit (Tiangen, Beijing, China). The RNA quantity and purity were checked by measuring the absorbance at 260 nm and the absorbance ratio of OD260/280 using a NanoVue UV‐Vis spectrophotometer (GE Healthcare Bio‐Science, Uppsala, Sweden), respectively. The RNA integrity was further verified by 1% agarose gel electrophoresis. The reverse transcription was carried out using a PrimeScript^®^ 1st Strand cDNA Synthesis Kit (Takara Biotechnology Dalian Co., Ltd., Dalian, China) and stored at –20 °C for future use.

Based on the transcriptome data of *T. cinnabarinus* (NCBI: SRA052165) and the genome database of *T. urticae* (http://bioinformatics.psb.ugent.be/orcae/overview/Tetur), sequence information of *UGT201D3* was acquired, and gene‐specific primers were designed and synthesized (Table S1) to directly amplify the complete open reading frame (ORF). Specific polymerase chain reaction (PCR) reaction was performed in a C1000™ Termal Cycler (Bio‐Rad, Hercules, CA, USA). The PCR conditions were set as follows: 3 min at 95 °C, followed by 35 cycles of 30 s at 95 °C, 30 s at 55–65 °C (depending on gene‐specific primers) and 60 s at 72 °C, then 10 min at 72 °C. Cloning and sequence analyses of the complete ORF of *UGT201D3* were repeated at least three times with different preparations of RNAs and further sequenced for confirmation (BGI, Beijing, China).

### Bioinformatic analysis of UGT201D3

The DNA sequence of *UGT201D3* was deduced by DNAMAN 5.2.2 (Lynnon Biosoft, San Ramon, CA, USA). The deduced sequence of amino acids was obtained through Primer Premier 5 (Version 5.00) and named by the UGT Nomenclature Committee (http://www.flinders.edu.au/medicine/sites/clinicalpharmacology/ugt-homepage.cfm) (Mackenzie *et al*., 2005). The multiple sequence alignment was performed online through ESPript 3.0 (http://espript.ibcp.fr/ESPript/cgi-bin/ESPript.cgi) (Robert *et al*., 2014). The signal peptide and transmembrane domain were predicted using SignalP 3.0 (http://www.cbs.dtu.dk/services/SignalP) (Bendtsen *et al*., 2004). The ExPASy proteomics tool (http://web.expasy.org/compute_pi/) was used to calculate the isoelectric point (pI) and molecular weight (MW) of *UGT201D3* (Bairoch, 1993). A phylogenetic tree was constructed by the maximum likelihood method using MEGA 5.05 software (Tamura *et al*., 2011). A total of 1000 bootstrap replications were used to test the phylogeny.

### Quantitative real‐time PCR (qPCR) of UGT201D3

In order to detect the expression level of *UGT201D3* in different developmental stages of the AbR strain, approximately 2000 eggs (1–2 days old), 1000 larvae (1 day old), 800 nymphs (the first nymph [1–2 days old]) and 200 female adults (3–5 day old) of mites were prepared for RNA extraction. To quantify this UGT gene expression in different strains, we collected 200 female adult mites in SS and AbR strains, respectively, for each sample. To examine the effect of abamectin exposure on the expression of *UGT201D3*, we used abamectin to treat adult female mites from SS and AbR strains (LC_10_ [SS: 0.03 mg/L, AbR: 1.0 mg/L], dissolved in acetone) for 6, 12 and 24 h, respectively. Mites treated with acetone were used as a control. The survivors were collected for RNA extraction. For the induction experiment, we also adopted the modified RCV method described as above. Each treatment contained three biological replicates and three technique replicates.

Primers of *UGT201D3* for qPCR study were designed by using primer 3.0 (http://frodo.wi.mit.edu/) (Rozen & Skaletsky, 2000) as shown in Table S1 along with a stable reference gene, ribosomal protein S18 (FJ608659) (Sun *et al*., 2010). Optimized PCR master mix (20 μL) contained the following components: 10 μL GoTaq® qPCR Master Mix (Promega, Madison, WI, USA), 1 μL diluted cDNA, 1 μL of each primer (10 μmol/L) and 7 μL ddH_2_O and the qPCR was performed on a Mx3000P thermal cycler (Agilent Technologies, Inc., Wilmington, NC, USA). The optimized thermal program was 95 °C for 2 min, then 40 cycles of denaturation at 95 °C for 15 s, 60 °C for 30 s. Finally, to ensure consistency and specificity of the amplified product, a melting curve analysis from 60 °C to 95 °C was applied. The qPCR experiments were performed according to minimum information for publication of qCR experiments (MIQE) guidelines (Garson *et al*., 2009). The relative expression of *UGT201D3* was analyzed using the $2^{-\Delta \Delta C_{T}}$ method (Livak & Schmittgen, 2001). The relative quantities of expression levels of *UGT201D3* in the SS and AbR strains were determined by an independent sample *t*‐test with a significance level of *P* < 0.05 in SPSS 19.0 (SPSS Inc., Chicago, IL, USA). The differences in *UGT201D3* expression levels among four developmental stages, *UGT201D3* expression levels before and after induction were analyzed by one‐way analyses of variance (ANOVA), followed by Tukey's Honestly Significant Difference (HSD) tests, with a significance level of *P* < 0.05 in SPSS 19.0. There were three biological replicates and three technique replicates to minimize intra‐experiment variation.

### RNAi of UGT201D3

RNAi was applied to further explore the toxicological function of *UGT201D3* in *T. cinnabarinus*. The green fluorescent protein (*GFP*) (ACY56286) gene was used as a negative control and diethylpyrocarbonate (DEPC)‐treated water was used as a blank control. The *UGT201D3* and the *GFP* genes were amplified by PCR using primers containing the T7 RNA polymerase promoter (Table S1). The double‐stranded RNAs (dsRNAs) were synthesized using TranscriptAid T7 High Yield Transcription Kit (Termo Scientifc, Lithuania, EU) on the basis of the manufacturer's instructions with the purified PCR products, and then purified the dsRNAs using the GeneJET RNA Purifcation Kit (Termo Scientifc, Lithuania, EU). The final dsRNAs were diluted with DEPC‐treated water to a concentration of 1000 ng/μL. The dsRNAs quantity and size were checked using a spectrophotometer and 1% agarose gel electrophoresis, respectively.

The RNAi was carried out according to a leaf‐disc feeding method described by Shi *et al*. (2015). After feeding for 48 h, the mites were collected to determine the reduction of transcription level of *UGT201D3* using qPCR. Meanwhile, in order to analyze whether there existed an off‐target effect, we also detected the expression levels of another two UGT genes (*UGT201B15* and *UGT201E2*) randomly from the same family with *UGT201D3* (Fig. 2). The qPCR primers are also listed in Table S1.

In this study, UGTs activity and bioassays with abamectin were tested after RNAi of *UGT201D3*. The 100–200 adult female mites at 48 h post‐feeding of *UGT201D3* dsRNA was collected for determination of UGTs activity. Three doses of abamectin LC_30_, LC_50_ and LC_70_ (the LC_30_, LC_50_ and LC_70_ values of abamectin were 0.10 mg/L, 0.25 mg/L and 0.61 mg/L for SS, 2.0 mg/L, 3.3 mg/L and 5.9 mg/L for the AbR strain) were applied for the bioassays. We also used the same procedure as described above. The RNAi knockdown efficiencies, mortality rates and specific activities of UGTs were analyzed by one‐way analysis of variance (ANOVA), followed by Tukey's HSD tests (*P*‐value < 0.05).

### Expression and purification of the UGT201D3

The specific primers of the original sequences of *UGT201D3* gene were designed (Table S1). According to reports (Fink, 1998), in the folding process of protein expression, the formation of inclusion bodies is related to the intermolecular interaction of the hydrophobic surface of the protein, and hydrophobic groups and β‐sheets are more likely to aggregate to form inclusion bodies. Then, online analysis of the hydrophilicity/hydrophobicity of the target protein via ProtScale (https://web.expasy.org/protscale/) shows that there are multiple hydrophobic regions in the target protein (Score > 0 is a hydrophobic region) (Fig. S1). Hence, to ensure that no active sites and conserved regions of the protein were removed, we designed primers (*UGT201D3q*) that remove 15 amino acids from the 5' hydrophobic region. Moreover, *Nde* I and *Eco*R I restriction enzyme cutting sites were incorporated into forward and reverse primers (Table S1). Then the target gene sequences and the expression vector pET‐28a were ligated together using T4 DNA‐ligase (Takara Biotechnology Dalian Co., Ltd., Dalian, China) after being linearized with the same enzymes, respectively. The products of ligation were transformed into Trans*5α* competent cells to identify positive clones. After that, the prokaryotic expression vectors pET‐28a‐*UGT201D3* and pET‐28a‐*UGT201D3q* were transformed into *E. coli* BL21 (DE3) strain and single colonies were cultured overnight with shaking at 37 °C. The grown bacterial cells with the proportion of 1% were inoculated into 500 mL Lysogeny broth‐kanamycin media shaken at 37 °C for 3–5 h until the OD600 reached 0.6–0.8. Then, IPTG was added to a final concentration of 0.1 mmol/L. The culture was subsequently incubated at 28 °C for 24 h at 180 r/min, and collected by centrifugation.

The cells were then harvested at 4 °C for 20 min by centrifugation at 4000 × *g* and re‐suspended in ice‐cold phosphate‐buffered saline (PBS) (0.04 mol/L, pH 7.4). Then the cells were disrupted by sonication (8 s, 150 W) on ice for 40 min and centrifuged at 9000 × *g* for 15 min at 4 °C. The supernatant was purified using a Ni^2+^‐NTA agarose gel column (Tiangen Biotech Co., Ltd., Beijing, China) with a linear imidazole gradient of 40–500 mmol/L in PBS (0.04 mol/L, pH 7.4), containing 0.5 mol/L NaCl. The quality of purified recombinant protein was determined by SDS‐PAGE (polyacrylamide gel electrophoresis) using a 5% (v/v) stacking gel and a 10% (v/v) resolving gel as well as western blotting with anti‐His tag monoclonal antibody.

### Enzyme activity and inhibition with acaricide measurements

The purified UGT201D3 protein concentration and the specific activity were measured using a microplate reader as described above.

Recombinant UGT201D3 protein (purified) kinetic variables (*K*_m_ [Michaelis constant] and *V*_max_ [maximum velocity]) were evaluated in the enzymatic assay from a double‐reciprocal plot of data obtained under assay conditions with different α‐naphthol concentrations (3–30 μmol/L). To investigate the substrate competitions of the recombinant enzyme between α‐naphthol and abamectin, the median inhibitory concentration (IC_50_) was determined at different concentrations of abamectin (0–100 μmol/L) with α‐naphthol concentration equal to the *K*_m_ of the recombinant UGT201D3 according to the enzymatic assay. For each combination of substrate and inhibitor at least three replicates were performed. In order to determine the type of inhibition, Dixon plot analysis were performed at three different concentrations of α‐naphthol (10, 20 and 40 μmol/L) and five different concentrations of abamectin (0, 30, 50, 80, 100 μmol/L). The experiments were repeated three times and results were analyzed with GraphPad prism 5 (GraphPad Software, Inc., San Diego, CA, USA).

### HPLC analysis of abamectin metabolism

The ability of purified UGT201D3 to metabolize abamectin was determined by HPLC. Tested reactions were performed by incubation of abamectin (10 mg/L) with UGT201D3 protein (150 μg) and UDP‐glucose (1 mmol/L) in 5% acetonitrile water solution with MgCl_2_ (10 mmol/L); the total volume was 1 mL. Two controls were designed: one is the same volume of the elution buffer and the other is the same as the protein treatment only except that the expression vector pET‐28a was not ligated with the *UGT201D3* gene. There were three biological replicates and three technique replicates. After incubating in a shaker at 28 °C, 180 r/min for 6 h, the reaction solution was transferred into a 15 mL centrifuge tube. The extraction of abamectin was conducted according to modified method of Zhang *et al*. (2012): 5 mL of ethyl acetate and 50 μL of 1% acetic acid aqueous solution were added to terminate the reactions and the samples were vortexed for 5 min. Then, after centrifuging for 5 min at 3500 r/min, the supernatant was totally transferred into a 100 mL flask. The extraction and centrifugation steps were repeated with fresh solvent. The extracts were combined and evaporated at 45 °C under vacuum (up to 100 mbar) with a rotary evaporator. Finally, the dry extract was redissolved to 1.0 mL with acetonitrile for HPLC analysis. Abamectin was separated on a SunFire C18 (5 μm, 150 mm × 4.6 mm) reverse phase analytical column (Waters Alliance 2695–2996). A mixture of acetonitrile and water (80 : 20, v/v) was used as mobile phase and the flow rate was 0.8 mL/min. The injection volume was 20 μL. Abamectin elution was monitored by changes in absorbance at 245 nm, and was quantified by peak integration. Data analyses were carried out using SPSS 19.0 (SPSS Inc., Chicago, IL, USA); the difference of the abamectin content between treatment and control was analyzed by an independent‐sample *t*‐test with a *P*‐value < 0.05.

## Results

### Activities of UGTs enzyme in SS and AbR strains of T. cinnabarinus

The specific activities of UGTs were 1.73 nmol/mg protein/min in AbR strain and 0.74 nmol/mg protein/min in SS, that is the activity of UGTs in AbR strain was 2.34‐fold of that in SS (Table 1), which suggested that the increase of UGT activity was probably correlated with *T. cinnabarinus*’s resistance against abamectin.

**Table 1:** The specific activity of uridine diphosphate‐glycosyltransferases (UGTs) in different strains of *Tetranychus cinnabarinus*

Strains	Specific activity (nmol/mg protein/min)
SS	0.74 ± 0.04
AbR	1.73 ± 0.29*

^*^Significant difference (*P *< 0.05).

SS, susceptible strain; AbR, abamectin‐resistant strain.

### Effect of synergist

After inhibiting the activity of UGTs in mites, the lethal effect of abamectin on resistant mites was significantly enhanced. The LC_50_ of the SS was reduced from 0.25 mg/L to 0.20 mg/L, and that in AbR strain was reduced from 3.51 mg/L to 1.51 mg/L. The toxicity of abamectin to SS changed without significance in the synergist test (the 95% CI of LC_50_ overlapped between the treatments with and without synergist), however, that in AbR strain significantly increased by 56.98% in the synergist treatment. The synergistic effect of UGTs inhibiter was significant in AbR while it was not in SS, which indicated that UGTs played an important role in abamectin resistance in *T. cinnabarinus* (Table 2).

**Table 2:** Toxicity of abamectin to SS and AbR strains with and without 5‐nitrouracil in *Tetranychus cinnabarinus*

Strains	Treatments	LC_50_ (mg/L) 95% CI	Slope (±SE)	χ^2^ *	Increased toxicity
SS	Abamectin	0.25 (0.20 – 0.34)	1.37 ± 0.21	2.25	–
	Abamectin + 5‐nitrouracil	0.20 (0.16 – 0.23)	1.70 ± 0.23	2.49	20.00%
AbR	Abamectin	3.51 (2.68 – 5.21)	2.27 ± 0.35	7.11	–
	Abamectin + 5‐nitrouracil	1.51 (1.33 – 1.70)	2.46 ± 0.30	1.88	56.98%

^*^Chi‐square testing linearity, *P *< 0.05.

SS, susceptible strain; AbR, abamectin‐resistant strain; LC_50_, lethal concentration at 50% mortality.

### Sequencing and annotation of UGT201D3 from T. cinnabarinus

**Figure 1:**
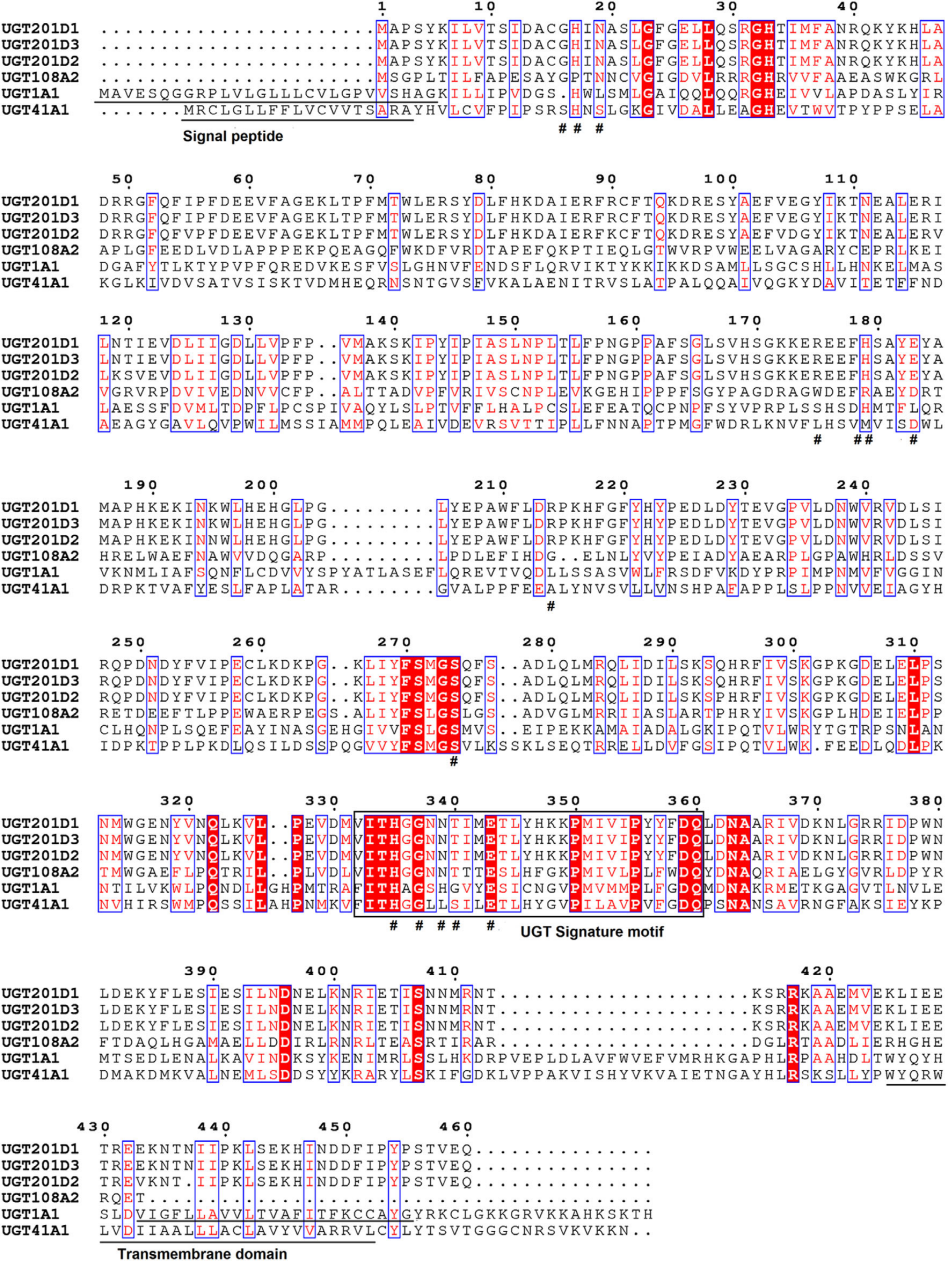
Alignment of amino acid sequence of *UGT201D3* in *Tetranychus cinnabarinas* and uridine diphosphate‐glycosyltransferases (UGTs) in other species. The UGT signature motif is boxed. The active sites predicted by the National Center for Biotechnology Information are be represented by #. (*UGT1A1* in human, *UGT41A1* in *Bombyx mori*, *UGT108A2* in bacteria, *UGT201D1* and *UGT201D2* in *Tetranychus urticae*).

**Figure 2:**
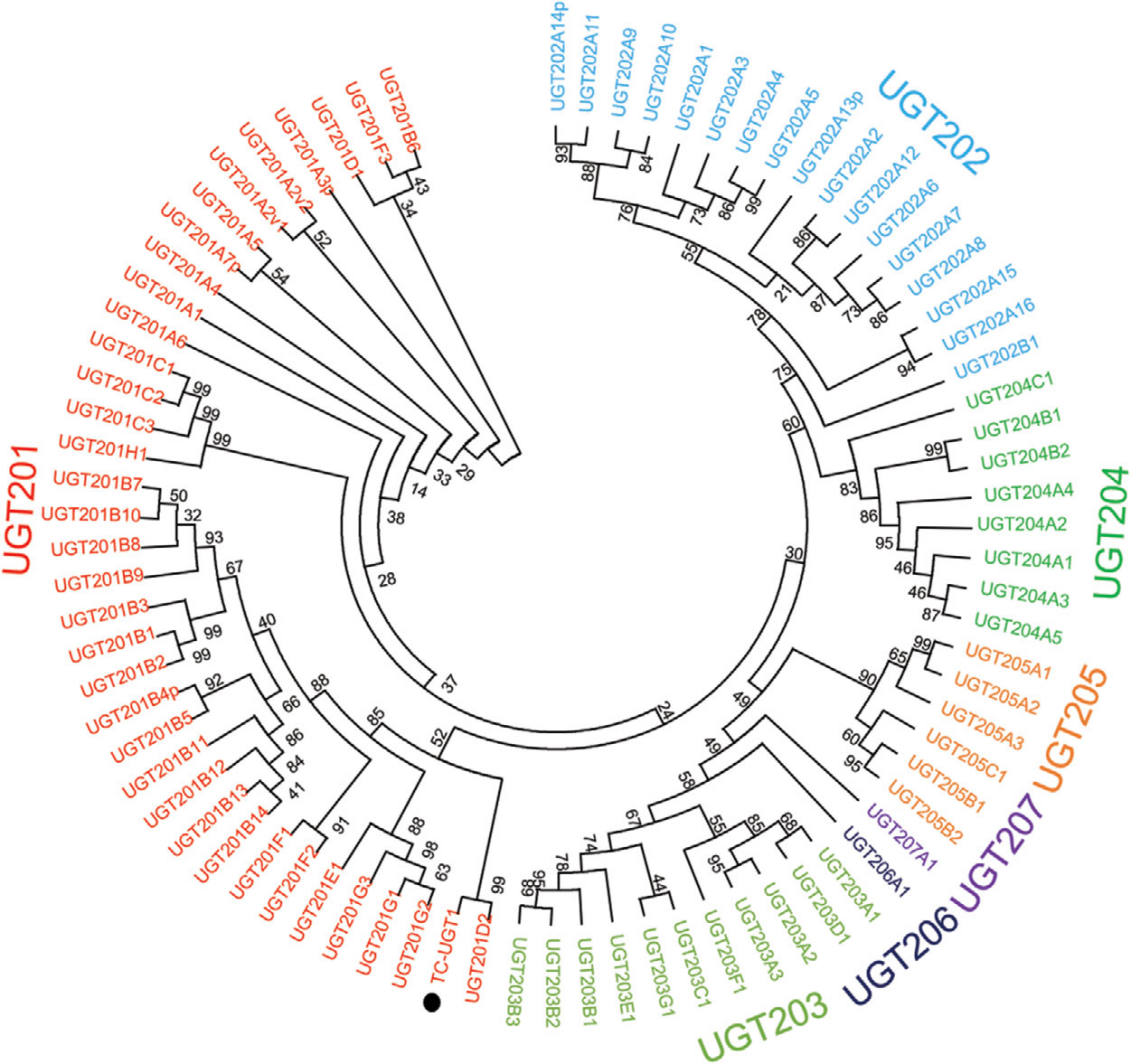
Phylogenetic relationship of *UGT201D3* with uridine diphosphate‐glycosyltransferase (UGT) family of *Tetranychus urticae*. The phylogram was generated using the maximum likelihood method in MEGA 5.05 and the inferred phylogeny was tested by bootstrap analysis with 1000 replications. The sequences used for constructing the tree are listed in Table S2. Filled circles indicate the newly cloned UGT gene of *T. cinnabarinus*. Filled triangles mean the other two UGT genes of *T. cinnabarinus* were used to analyze whether there are off‐target effects in RNA interference (RNAi).

From the previous microarray (GEO: GSE70824) results, we found an UGT gene over‐expressed in AbR strain. Then, this gene containing complete ORFs was obtained according to the transcriptome data of *T. cinnabarinus* and the genome of *T. urticae*. It was named as *UGT201D3* by the UGT Nomenclature Committee. Sequence information has been submitted to GenBank and the accession number is KX905079. The ORF of *UGT201D3* is 1383 bp, encoding 460 amino acid residues. According to the prediction results, *UGT201D3* gene has no signal peptide and transmembrane domain. A characteristic signature sequence was identified in its C‐terminal, which contained the conserved amino acids: [FAV]‐[LIVMF]‐[TS]‐[HQ]‐[SGAC]‐G‐X(2)‐[STG]‐X(2)‐[DE]‐X(6)‐P‐[LIVMFA]‐[LIVMFF]‐X(2)‐P‐[LMVFIQ]‐X(2)‐[DE]‐Q (amino acids in square brackets can be arbitrary, and X represents any amino acid). The signature motif has been implicated in binding the UDP‐sugar (Mackenzie *et al*., 2005) (Fig. 1). The molecular weight of the predicted protein and estimated isoelectric point (pI) value were 53.49 kDa and 5.74, respectively. The phylogenetic analyses were performed by MEGA 5.05 with the maximum likelihood method on the basis of the deduced amino acid sequences of *UGT201D3* and all UGT family genes of *T. urticae* (Ahn *et al*., 2014). The phylogenetic tree showed that the *UGT201D3* shared 98% identity with *UGT201D2* (tetur04g04350) from *T. urticae* (Fig. 2).

### Specific expression of UGT201D3 in different developmental stages, strains and abamectin‐induction treatment

**Figure 3:**
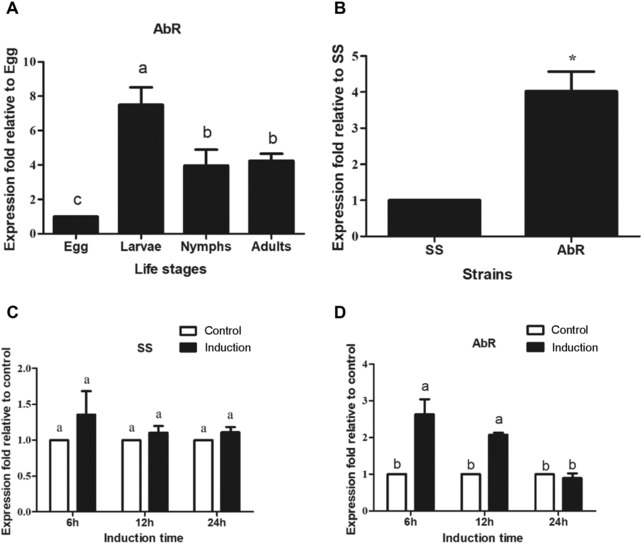
Quantitative polymerase chain reaction analysis of the expression of *UGT201D3* in *Tetranychus cinnabarinus*. (A) The expression of *UGT201D3* in different life stages in abamectin‐resistant strain (AbR); (B) the expression of *UGT201D3* in different strains (3–5 days old female adult mites); (C, D) the expression of *UGT201D3* to abamectin exposure in the susceptible (SS) and AbR strains (3–5 days old female adult mites). The error bars represent the standard deviation of the mean of three independent replicates. Asterisks on the error bars show significant differences between the SS and AbR strains (*P *< 0.05). Different letters on the error bars show significant differences among different developmental stages or between the abamectin induction and the control (*P *< 0.05).

To clarify the characteristics of *UGT201D3* gene expression in different developmental stages of *T. cinnabarinus*, qPCR analysis was performed on the cDNA from eggs, larvae, nymphs and adults of the AbR strain. The relative expression level of *UGT201D3* in larvae, nymphs and adults were 7.36‐, 4.16‐ and 4.17‐fold higher than that in eggs (Fig. 3A). Identification of the differences in the levels of expression of *UGT201D3* between the SS and AbR strains showed that *UGT201D3* was significantly up‐regulated to 4.02‐fold in the AbR strain compared with SS (Fig. 3B). The results of abamectin induction experiment revealed that there were no significant differences between treatments and control in SS, which suggested that the expression of *UGT201D3* cannot be induced in SS. By contrast, the transcript of *UGT201D3* was significantly increased (2.63‐ and 2.10‐fold, 6 h and 12 h later of abamectin‐exposure, respectively) in the AbR strain (Fig. 3C, D).

### Functional analysis of UGT201D3 by RNAi

**Figure 4:**
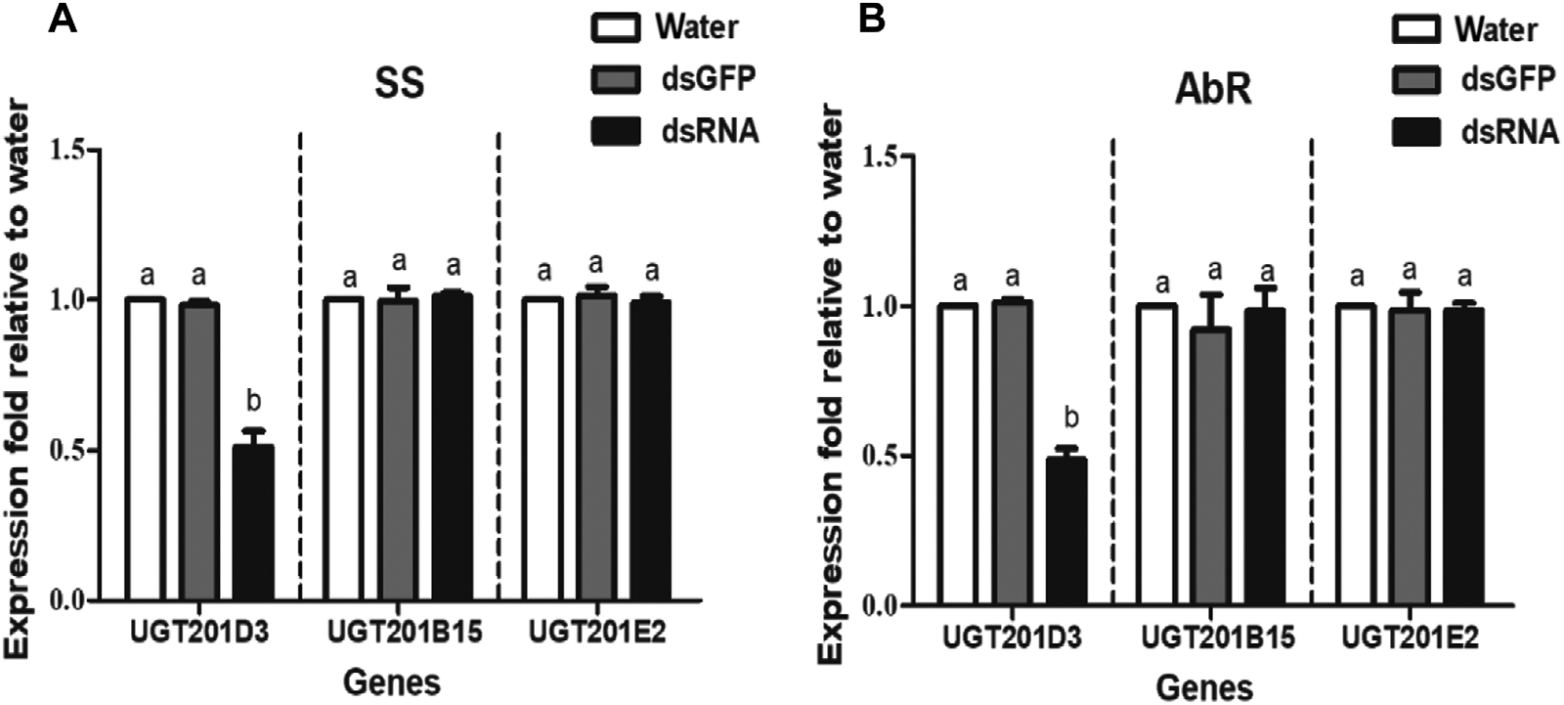
Quantitative polymerase chain reaction analysis of the expression of *UGT201D3* after RNA interference (RNAi) in the susceptible (SS) and abamectin‐resistant (AbR) strains of *Tetranychus cinnabarinus*, relative to expression in the water control. *UGT201B15* and *UGT201E2* are another two uridine diphosphate‐glycosyltransferase (UGT) genes used to analyze whether there exist off‐target effects in the RNAi. The error bars represent the standard deviation of the mean of three independent replicates. Different letters above bars indicate significant differences among treatments (*P *< 0.05).

After RNAi, the relative expression levels of mRNA were detected. The results showed that the transcript levels of *UGT201D3* in the SS and AbR strains were significantly decreased by 48.82% and 51.25% compared with the controls, respectively (Fig. 4). Meanwhile, the qPCR data also showed that the expression levels of the other two UGT genes (*UGT201B15* and *UGT201E2*) were not affected by RNAi neither in SS nor in AbR (Fig. 4). These results demonstrated that the transcript of *UGT201D3* was successfully knocked down with no off‐target effect.

**Figure 5:**
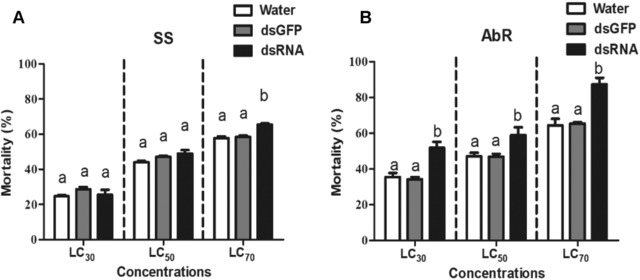
Mortality after RNA interference (RNAi) of *UGT201D3* in the susceptible (SS) and abamectin‐resistant (AbR) strains of *Tetranychus cinnabarinus*. The error bars represent the standard deviation of the mean of three independent replicates. Different letters above bars indicate significant differences among treatments (*P *< 0.05). LC_30_: lethal concentration at 30% mortality. LC_50_: lethal concentration at 50% mortality. LC_70_: lethal concentration at 70% mortality.

Subsequently, the specific activities of UGT enzymes were detected after decreasing the expression of *UGT201D3*. The activities of UGTs decreased significantly about 1.68‐fold and 2.72‐fold in SS and AbR strains (Table 3). Furthermore, the mortalities increased significantly by 16.42%, 11.94% and 22.99%, respectively, when treated with three concentrations of abamectin (LC_30_, LC_50_ and LC_70_ of abamectin) in the AbR strain. However, there were no significant differences of the mortalities between the treatment and controls in SS except for the mortality of LC_70_ of abamectin (Fig. 5). The increased mortalities in AbR implied that down‐regulation of *UGT201D3* could decrease the abamectin‐resistance in *T. cinnabarinus*.

**Table 3:** The specific activity of uridine diphosphate‐glycosyltransferases (UGTs) after dsRNA feeding in different strains of *T. cinnabarinus*

	Specific activity (nmol/mg protein/min)*	
Treatments	SS	AbR
DEPC‐water	0.541 ± 0.081 a	1.727 ± 0.171 a
dsGFP	0.529 ± 0.036 a	1.716 ± 0.235 a
dsRNA	0.321 ± 0.029 b	0.634 ± 0.101 b

^*^Values within a column followed by different letters are significantly different (*P *< 0.05).

SS, susceptible strain; AbR, abamectin‐resistant strain; dsRNA, double‐stranded RNA; DEPC, diethylpyrocarbonate; dsGFP, double‐stranded green fluorescent protein.

### Expression of UGT201D3 in E. coli

**Figure 6:**
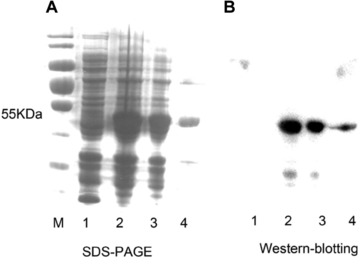
SDS‐PAGE (A) and western blotting (B) analysis of recombinant UGT201D3 expression in *Escherichia coli* (DE3). Lane M: protein markers; Lane 1: PET‐28a with isopropyl b‐D‐1‐thiogalactopyranoside (IPTG) induction; Lane 2: PET‐28a‐*UGT201D3q* with IPTG induction; Lane 3: soluble protein; Lane 4: purified by Ni^2+^‐NTA.

To facilitate functionally expressing *UGT201D3*, the original sequence and N‐terminal modification sequence of *UGT201D3* (pET‐28a‐*UGT201D3* and pET‐28a‐*UGT201D3q*) were expressed in *E. coli*. Induction of the pET‐28a‐*UGT201D3* and pET‐28a‐*UGT201D3q* with 0.1 mmol/L IPTG for 24 h at 28 °C in BL21 (DE3) cells resulted in good levels of protein production. However, only the pET‐28a‐*UGT201D3q* obtained the majority of soluble protein in the supernatant. After purification using a Ni^2+^‐NTA agarose gel column, the recombinant UGT201D3 protein was analyzed by SDS‐PAGE and western blotting. These results showed that the recombinant UGT201D3 was well purified. The actual molecular weight was consistent with the predicted molecular mass (51.89 KDa) (Fig. 6).

**Figure 7:**
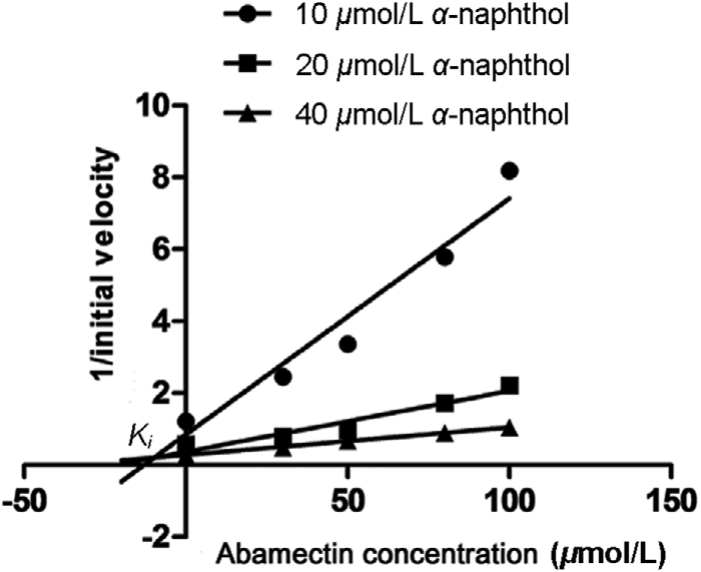
Dixon plot analysis for the inhibition of α‐naphthol conjugating activity of UGT201D3 by different abamectin concentrations. Three concentrations of α‐naphthol (10, 20 and 40 μmol/L) and five concentrations of abamectin (0, 30, 50, 80, 100 μmol/L) were used and data are means of three replicates ± SD. Analysis denoted a competitive type of inhibition and the inhibition constant (*K_i_*) was determined as 9.9 ± 6.2 μmol/L.

The enzyme activity of purified UGT201D3 was studied using α‐naphthol as substrate and UDP‐glucose as a sugar donor. The specific activity of the recombinant UGT201D3 was 2.81 ± 0.43 nmol/mg/protein/min. The values of *K*_m_ and *V*_max_ were 22.73 ± 5.87 μmol/L and 11.08 ± 2.77 nmol/mg/protein/min (Table 4), respectively. The activity of UGT201D3 was inhibited by abamectin and the IC_50_ was determined at 57.50 ± 3.54 μmol/L (Table 5). Dixon plot analysis showed that the inhibition was competitive for the three linear curves (three different concentrations of α‐naphthol) crossed above the *x* axis and the inhibition constant (*K_i_*) was determined at 9.9 ± 6.2 μmol/L (Fig. 7).

**Table 4:** The specific activity and kinetic variables of recombinant UGT201D3

Samples	Specific activity (nmol/mg pro/min)	*K*_m_ (μmol/L)	*V*_max_ (nmol/mg pro/min)
PET‐28a vector	–	–	–
Recombinant UGT201D3	2.81 ± 0.43	22.73 ± 5.87	11.08 ± 2.77

*K*_m_, Michaelis constant; *V*_max_, maximum velocity.

**Table 5:** Ability of abamectin to inhibit α‐naphthol glycosylation catalyzed by UGT201D3

Acaricides	Chemical type	IC_50_ (μmol/L)
Abamectin	Macrolide	57.50 ± 3.54

IC_50_, inhibitory concentration at 50%.

### Assessing the capability of UGT201D3 to deplete abamectin

To further confirm whether recombinant UGT201D3 protein can metabolize or deplete abamectin directly, catalytic activity was initially assessed by measuring substrate depletion using HPLC. The average recoveries of abamectin at three spiked levels were 93.72%–107.30% with the relative standard deviation ranging from 2.54% to 7.16%, which indicated the developed method was valid and reliable for residual analysis of abamectin in the enzyme metabolism study (Table S3). Compared to the elution buffer, the protein expressed by the empty vector pET‐28a cannot deplete abamectin. Compared with the empty vector, the addition of recombinant UGT201D3 protein detected a significant decrease in the abamectin content, in which the desired new metabolite was not detected. The results showed that the rate of depletion for abamectin was 15.77% ± 3.72% when treated with 150 μg recombinant UGT201D3 for 6 h (Table 6).

**Table 6:** *In vitro* depletion of abamectin by recombinant UGT201D3

		Abamectin content (mg/mL)		
Protein	Protein content (μg/mL)	Initial	Incubation 6 h	Depleting rate (%) *, †
Recombinant UGT201D3	150	10	8.42 ± 0.37	15.77% ± 3.72% b
PET‐28a vector	–	10	9.93 ± 0.13	0.67% ± 1.30% a
Elution buffer	–	10	9.92 ± 0.16	0.80% ± 1.64% a

^*^Values within a column followed by different letters are significantly different (*P *< 0.05).

^†^Depleting rate = (initial content of abamectin – abamectin content after incubating 6 h) / initial content of abamectin × 100.

## Discussion

Abamectin has been widely used as an insecticide/acaricide for more than 30 years because of its superior bioactivity against insect and mite pest (Xu *et al*., 2017). So far, many arthropod species, such as *P. xylostella*, *T. urticae* and *T. cinnabarinus*, have evolved various levels of resistance to abamectin worldwide (Leeuwen *et al*., 2010). The inheritance of abamectin‐resistance was reported in *T. cinnabarinus* (He *et al*., 2009a), *P. xylostella* (Liang *et al*., 2003) and other insects or mites (Clark *et al*., 1992; Ferreira *et al*., 2015), which was controlled by multiple genes and the degree of dominance was incomplete recessive. Polygenic resistance was confirmed by multiple abamectin‐resistant mechanisms documented in insects and mites. Mutations in the target site of glutamate‐gated chloride channels conferred resistance to abamecctin in *T. urticae* and *P. xylostella* (Kwon *et al*., 2010; Dermauw *et al*., 2012; Wang *et al*., 2016). Considerable studies showed that CarEs, mixed function oxidases (MFOs) and glutathione‐*S*‐transferases (GSTs) were the most important enzymes correlated with abamectin‐metabolic resistance in arthropods (Liang *et al*., 2001; He *et al*., 2009b; Deokho *et al*., 2010; Riga *et al*., 2014). The increased expression of P‐glycoprotein (P‐gp) also contributed to abamectin‐resistance in *Drosophila melanogaster* (Luo *et al*., 2013) and *T. cinnabarinus* (Xu *et al*., 2016), respectively. Besides the above‐mentioned mechanisms, here we have elucidated another mechanism of abamectin resistance in *T. cinnabarinus*, which is mediated by the UGTs.

In recent years, the UGTs, which can glycosylate toxins, like plant secondary metabolites, has been identified to be a detoxification enzyme in insects. For example, the larvae of the autumnal moth, *Epirrita autumnata*, can detoxify toxic flavonoid aglycones present in birch leaves via glycosylation (Salminen *et al*., 2015). Krempl and colleagues proved that UGTs could metabolize gossypol partially via glycosylation in *H. armigera* and *H. virescens*, which might be a crucial step in gossypol detoxification (Krempl *et al*., 2016). From transcriptome analysis, UGT genes were subsequently found to be over‐transcribed in resistant populations, for instance in DDT‐resistant *D. melanogaster* (Pedra *et al*., 2004), carbamate‐resistant *Myzus persicae* (Silva *et al*., 2012), neonicotinoid‐resistant *Bemisia tabaci* (Yang *et al*., 2013) and permethrin‐induced *Anopheles gambiae* (Vontas *et al*., 2005). Recently, some UGT genes were reported have function‐mediating resistance against imidacloprid (Kaplanoglu *et al*., 2017) and chlorantraniliprole (Li *et al*., 2017) with the RNAi method. In *Caenorhabditis elegans* (Laing *et al*., 2010) and *Haemonchus contortus* (Vokřál *et al*., 2012) the covalent linkage of glucose with benzimidazole anthelmintics was catalyzed by UGTs. Moreover, it was also proved that UGTs of *H. contortus* were able to detoxify naphthalophos, an organophosphate, and they were also involved in anthelmintic‐resistance (Kotze *et al*., 2014). All these findings implied the potential roles of UGTs in the detoxification of insecticides, and UGTs could mediate metabolic resistance, whereas no further and direct evidence were documented to confirm the interaction between UGT gene products and insecticides until the present study.

It was reported that 5‐nitrouracil (5‐NU), a specific inhibitor of UGTs, could increase the toxicity of insecticides to *P. xylostella* (Li *et al*., 2017) and *H. contortus* (Kotze *et al*., 2014) by inhibiting UGT activity. In the current study, both enzyme activity assay and enzyme inhibitor bioassay indicated that elevating activity of UGTs was associated with abamectin resistance in *T. cinnabarinus*. The qPCR analysis in our study showed that the expression of *UGT201D3* was higher in AbR strain than that in SS, and the expression of *UGT201D3* was increased significantly in AbR after stimulation with abamectin, which implied that *UGT201D3* gene could have an important role conferring abamectin resistance in *T. cinnabarinus*. Similarly, Vontas *et al*. (2005) found that UGT genes emerged with different transcriptional levels in the permethrin‐resistant strain and showed elevated mRNA levels after permethrin exposure in *A. gambiae*. It has also been reported that *UGT40R3* and *UGT46A*6 could be induced by deltamethrin in antennae of *Spodoptera littoralis* (Bozzolan *et al*., 2014). RNAi has been widely used to investigate gene function in insects and has proved to be an effective technique with high specificity (Zhu, 2013). After silencing the expressions of two UGT genes (*UGT1* and *UGT2*) via RNAi in the imidacloprid‐resistant strain of *L. decemlineata*, susceptibility of resistant beetles to imidacloprid significantly increased, indicating that the UGT genes were related to imidacloprid resistance (Kaplanoglu *et al*., 2017). Likewise, similar RNAi results in *P. xylostella* revealed that *UGT2B17* was involved in the detoxification of chlorantraniliprole, and its over‐expression played an important role in chlorantraniliprole‐resistance in *P. xylostella* (Li *et al*., 2017). In our study, after knocking down the *UGT201D3* via RNAi, the mortalities increased dramatically and the activity of UGTs decreased in the AbR strain, indicating that *UGT201D3* could be an important factor involved in abamectin resistance in *T. cinnabarinus*. It was interesting to note that the mortalities under LC_70_ of abamectin in the treatment was obviously increased comparing that in the controls in SS. This might be another evidence that *UGT201D3* was important in abamectin detoxification and abamectin‐resistance formation in *T. cinnabarinus* because it worked and resisted high concentrations of abamectin in SS.

Prokaryotic expression is commonly used in the study of gene function in insects or mites. However, due to the inherent characteristics of the expression system of *E. coli*, there are deficiencies. In practice, the target gene is often modified, including the N‐terminal of the gene. For example, the signal peptide of the P450 gene is removed, and the previous eight amino acids are replaced; or a certain codon of the gene is mutated to facilitate expression (Barnes *et al*., 1991). It has been found that comparing the N‐terminal modified human P450 expressed in *E. coli* and the same N‐terminal modified enzyme expressed by insect cells, it was confirmed that the N‐terminal modification did not affect the catalytic properties of P450 (Yamazaki *et al*., 1999). In addition, the inclusion of hydrophobic groups and β‐sheets in the peptide chain made it easier to form inclusion bodies (Fink, 1998). Mutating immunoglobulin hydrophobic groups into hydrophilic groups could significantly reduce the formation of inclusion bodies in the *E. coli* expression system (Nieba *et al*., 1997). Therefore, in order to clarify the function of *UGT201D3* gene expressed in *T. cinnabarinus* in abamectin resistance, this study uses the *E. coli* expression system to express it *in vitro*. However, when the original sequence of the gene was expressed, only the inclusion body was obtained. So, we modified the gene via removing the N‐terminal hydrophobic region without affecting the active site and the conserved region. Fortunately, the recombinant soluble protein with UGT activity was obtained. The inhibition test and Dixon plot analysis revealed abamectin could be the substrates of UGT201D3 protein. Subsequently, the HPLC analysis showed that abamectin could be depleted by the recombinant UGT201D3 protein, which provided direct evidence that *UGT201D3* played a role in abamectin resistance in *T. cinnabarinus*. However, we could not say that abamectin was metabolized or degraded by UGT201D3 protein considering the general function of UGTs (glycosylation) and the fact that the novel products from interaction were not detected under the current conditions (Fig. S2). Before the current study about UGTs, we previously documented that the resistance against abamectin in *T. cinnabarinus* was controlled by polygenes, the detoxifying enzymes, such as CarEs, MFOs, GSTs and P‐gp contributing to abamectin resistance (He *et al*., 2009a, 2009b; Xu *et al*., 2016). However, it is hard to state which enzyme is the major factor that mediates abamectin resistance in *T. cinnabarinus* based on existing results, and systematic and comparative studies should be explored in the future.

In summary, all of the studies we have done demonstrated that *UGT201D3* contributed to abamectin‐resistance formation in *T. cinnabarinus*. This is the first time it has been directly proved that UGT gene up‐regulation can mediate abamectin‐resistance through detoxifying abamectin. These findings will shed light on our understanding of UGT gene function in arthropods and the mechanisms of abamectin‐resistance formation in *T. cinnabarinus*. It would be reasonable that the similar resistant mechanism may exist in resistant strains of *T. cinnabarinus* in the filed because the UGT genes mediating resistance have been confirmed in resistant field insects, such as *L. decemlineata* (Kaplanoglu *et al*., 2017) and *P. xylostella* (Li *et al*., 2017). Furthermore, Pedra *et al*. (2004) reported that the field and laboratory DDT‐resistant *Drosophila* genotypes shared much commonalities of over‐transcribed genes and concluded that similar resistance mechanisms may exist between laboratory‐ and field‐selected DDT‐resistant fly lines, which further strengthened the possibility of our inference.

## Discloure

The authors declare no conflict of interest.

## Supporting information


**Table S1**. Primers used for cloning, RNAi and qPCR analysis.
**Table S2**. Sequences used for phylogenetic analysis.
**Fig. S1**. The analysis of amino acid hydrophilicity/hydrophobicity of *UGT201D3*. Score > 0 is a hydrophobic region. Inside the red box is 15 amino acids removed.
