# Supplementary material for: Functional analysis of UGT201D3 associated with abamectin resistance in Tetranychus cinnabarinus (Boisduval)
Source: Insect Sci. 2018 Sep 26;27(2):276–91. doi: 10.1111/1744-7917.12637 (PMC7379272; doi:10.1111/1744-7917.12637)
Supplement: Supplementary file 1 — Table S1. Primers used for cloning, RNAi and qPCR analysis. Table S2. Sequences used for phylogenetic analysis. Fig. S1. The analysis of amino acid hydrophilicity/hydrophobicity of UGT201D3. Score > 0 is a hydrophobic region. Inside the red box is 15 amino acids removed. [file INS-27-276-s001.docx]

Table S1. Primers used for cloning, RNAi and qPCR analysis

| Genes | Primer Function | | Primer sequences (5' to 3') |
| --- | --- | --- | --- |
| *UGT201D3* | Cloning full length | | F: ATGGCGCCATCATACAAAAT |
|  |  |  | R: ATCAACTGTTGAACAATGA |
|  | RNAi | | F:taatacgactcactatagggGACTTAATCATTGGCGATCT |
|  |  |  | R:taatacgactcactatagggTCAACTCGAACCCAATTATC |
|  | qPCR | | R: CTCGATCCTTCTGGGTGAAA |
|  |  |  | F: TGCATCTCTTGGTTTTGGTG |
|  | Heterologous expression | *UGT201D3* | F: GGAATTCCATATGGCGCCATCATACAAAAT  R: CCGGAATTCTCATTGTTCAACAGTTGAT |
|  |  | *UGT201D3q* | F：GGAATTCCATATGGGACATATCAATGCATC  R：CCGGAATTCTCATTGTTCAACAGTTGAT |
| *UGT201B15* | qPCR | | F: CCACCATTCTCTGGTTACTC  R: GGCTGGGTTGAACTTGACAT |
| *UGT201E2* | qPCR | | F: TGCAGCATCCAAATCTTGTC |
|  |  |  | R: TGTTGTTGCCTCCATGTGTT |
| *GFP* | RNAi | | F:taatacgactcactatagggCAGTTCTTGTTGAATTAGATG  R:taatacgactcactatagggTTTGGTTTGTCTCCCATGATG |

Underlines indicate Nde I and EcoR I restriction enzyme cutting sites

Table S2. Sequences used for phylogenetic analysis

| Genes | Gene ID | Genes | Gene ID |
| --- | --- | --- | --- |
| *UGT201D2* | tetur04g04350 | *UGT202A13p* | tetur30g02050 |
| *UGT201D1* | tetur04g04300 | *UGT202A1* | tetur15g00340 |
| *UGT201E1* | tetur05g05710 | *UGT202A3* | tetur22g00310 |
| *UGT201G2* | tetur12g00360 | *UGT202A4* | tetur22g00330 |
| *UGT201G1* | tetur08g07460 | *UGT202A5* | tetur22g00350 |
| *UGT201G3* | tetur02g10390 | *UGT202A2* | tetur22g00270 |
| *UGT201F2* | tetur06g02410 | *UGT202A12* | tetur30g00390 |
| *UGT201F3* | tetur06g02430 | *UGT202A6* | tetur22g00360 |
| *UGT201F1* | tetur02g01310 | *UGT202A7* | tetur22g00380 |
| *UGT201B14* | tetur07g06450 | *UGT202A8* | tetur22g00420 |
| *UGT201B13* | tetur07g06430 | *UGT202A15* | tetur22g00440 |
| *UGT201B12* | tetur07g06420 | *UGT202A16* | tetur22g00970 |
| *UGT201B11* | tetur07g06390 | *UGT203A1* | tetur01g07060 |
| *UGT201B5* | tetur04g07770 | *UGT203D1* | tetur10g05770 |
| *UGT201B6* | tetur04g07780 | *UGT203A2* | tetur04g02350 |
| *UGT201B4p* | tetur04g07710 | *UGT203A3* | tetur06g06100 |
| *UGT201B10* | tetur05g05060 | *UGT203F1* | tetur36g00340 |
| *UGT201B9* | tetur05g05050 | *UGT203C1* | tetur05g05090 |
| *UGT201B8* | tetur05g05030 | *UGT203G1* | tetur36g01060 |
| *UGT201B7* | tetur05g05020 | *UGT203B1* | tetur09g00220 |
| *UGT201B3* | tetur04g07630 | *UGT203B2* | tetur09g01650 |
| *UGT201B2* | tetur01g05700 | *UGT203B3* | tetur09g01660 |
| *UGT201B1* | tetur01g05690 | *UGT203E1* | tetur16g02300 |
| *UGT201H1* | tetur08g03000 | *UGT204A1* | tetur02g03300 |
| *UGT201C3* | tetur05g04690 | *UGT204A2* | tetur05g00060 |
| *UGT201C2* | tetur05g04680 | *UGT204A3* | tetur05g00070 |
| *UGT201C1* | tetur01g11870 | *UGT204A5* | tetur05g00090 |
| *UGT201A6* | tetur19g00440 | *UGT204A4* | tetur05g00080 |
| *UGT201A4* | tetur05g09325 | *UGT204B1* | tetur02g09830 |
| *UGT201A7p* | tetur21g01400 | *UGT204B2* | tetur02g09850 |
| *UGT201A5* | tetur08g00190 | *UGT204C1* | tetur11g06460 |
| *UGT201A8* | tetur184g00030 | *UGT205A1* | tetur11g01230 |
| *UGT201A3p* | tetur02g02770 | *UGT205A2* | tetur11g01250 |
| *UGT201A2v1* | tetur60g00080 | *UGT205A3* | tetur32g01240 |
| *UGT201A2v2* | tetur02g02480 | *UGT205C1* | tetur11g01830 |
| *UGT201A1* | tetur01g03820 | *UGT205B1* | tetur32g01230 |
| *UGT202B1* | tetur10g02090 | *UGT205B2* | tetur32g01250 |
| *UGT202A14p* | tetur139g00010 | *UGT206A1* | tetur08g05390 |
| *UGT202A11* | tetur22g00510 | *UGT207A1* | tetur08g02490 |
| *UGT202A9* | tetur22g00460 | *UGT201B15* | KY355635 |
| *UGT202A10* | tetur22g00480 | *UGT201E2* | KY355636 |

Table S3 Recovery of the analytical method for the measurement of abamectin (n=3)

| Spiked levels(mg/L) | Recovery (%)^a*^ | RSD(%)^b^ |
| --- | --- | --- |
| 0.1 | 97.37±6.97 | 7.16 |
| 0.5 | 93.72±4.80 | 5.12 |
| 5 | 107.30±2.72 | 2.54 |

^a^Values represent the mean ± standard deviation (SD).

^b^RSD represents the relative standard deviation.

^*^The recoveries of three concentrations were in 70-110% range, which meant the HPLC-method was valid and reliable according to EU standard (Guidance document on pesticide residue analytical methods, EUROPEAN COMMISSION, SANCO/825/00 rev. 8.1, 16/11/2010, Page 10, Line 278).

Figure S1


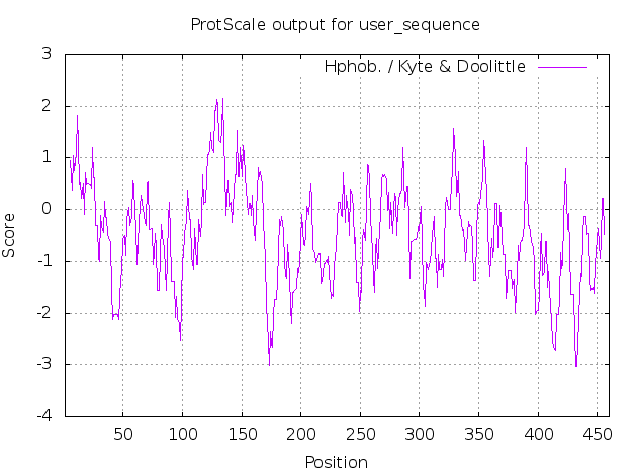


Fig. S1 The analysis of amino acid hydrophilicity / hydrophobicity of *UGT201D3.* Score>0 is a hydrophobic region. Inside the red box is 15 amino acids removed.

Figure S2

A

B

retention time

C

Fig S2. HPLC chromatograms of metabolism of abamectin by UGT201D3. (A) Standard sample of abamectin (retention time is 16.3 min), (B) after incubating abameictin with PET-28a, (C) after incubating abameictin with UGT201D3.
